# Supplementary material for: How can managed entry agreements contribute to coverage decisions in Latin America?
Source: Int J Technol Assess Health Care. 2024 Dec 2;40(1):e65. doi: 10.1017/S0266462324000527 (PMC11703624; doi:10.1017/S0266462324000527)
Supplement: García Martí et al. supplementary material 1 — García Martí et al. supplementary material [file S0266462324000527sup001.docx]

**Annex I**

**Participant List**

We thank all Policy Forum participants for their interest, involvement, and contributions.

**NON-PROFIT ORGANIZATIONS**

1. Silvina Beatriz Benchetrit / Ministry of Health GCABA / ARGENTINA

2. Manuel Donato / Ministry of Health Argentina / ARGENTINA

3. Luciene Bonan / National Committee for Technology Incorporation (CONITEC) / BRAZIL

4. Silvana Kelles / Unimed / BRAZIL

5. Felipe Vera Chandia / Ministry of Health Chile / CHILE

6. Adriana María Robayo García / Institute for Health Technology Assessment (IETS) /

COLOMBIA

7. Lizbeth Alexandra Acuña Merchan / Cuenta de Alto Costo / COLOMBIA

8. Hugo Marín Piva / Caja Costarricense de Seguro Social (CCSSS) / COSTA RICA

9. Carlos Diaz Huerta / Instituto Mexicano Seguro Social (IMSS) / MEXICO

10. Verónica Gallegos / Centro Nacional de Excelencia Tecnológica en Salud (CENETEC) /

MEXICO

11. Pedro Galvan / Instituto de Investigaciones en Ciencias de la Salud (IICS) / PARAGUAY

12. Lely Solari / Instituto Nacional de Salud (INS) / PERU

13. Andrea Gimenez / Ministry of Health Uruguay / URUGUAY

14. Graciela Fernandez / Fondo Nacional de Recursos (FNR) / URUGUAY

15. Yesenia Diaz / Superintendencia de Salud y Riesgos Laborales (SISALRIL) / REPÚBLICA

DOMINICANA

16. Ricardo Ruano / Ministry of Health El Salvador / EL SALVADOR

17. Natalia Messina / Ministry of Health de Argentina / ARGENTINA

18. Héctor Castro / Pan American Health Organization, World Health Organization /

COLOMBIA

19. Pilar Nehuelhal / Ministry of Health Chile / CHILE

**PARTICIPANTES FROM FOR-PROFIT ORGANIZATIONS**

1. Daniel Campos, ABBVIE

2. Rogerio Afif, ABBVIE

3. Mohit Jain, BioMarin

4. José Thomas, BioMarin

5. Javier Garcia, Bristol Myers Squibb

6. Arely Lemus, Bristol Myers Squibb

7. Fabian Ochoa, Edwards Lifesciences

8. Alma Delia Carbajal, Eli Lilly

9. Joice Valentim, F. HOFFMANN-LA ROCHE AG

10. Virginia Becerra, Roche International Ltd.

11. Natalia Tassara, Johnson and Jonhson

12. María Camila Bustos, Johnson and Jonhson

13. Daniela Paredes, Medtronic

14. Juan Valencia, Medtronic

15. Cintia Parellada, MERCK & CO

16. Diego Guarin, MERCK & CO

17. Nicola Romanello, PFIZER LIMITED

18. Mac Mullen, Mercedes, PFIZER LIMITED

19. Rafael do Prado Souza, Sanofi

20. Antonio Dos Santos, Sanofi

**PATIENT REPRESENTATIVES**

1. Cecilia Rodriguez / Ministry of Health, Chile – Area of Citizen Participation / CHILE

2. Alejandro Andrade / Chilean Federation of Rare Diseases - FECHER / CHILE

3. Virginia Llera / Geiser Foundation / ARGENTINA

4. Eva María Ruiz de Castilla / Latin America Patient Academy LAPA

**INVITED SPEAKER**

1. Pilar Pinilla-Dominguez / NICE / UNITED KINGDOM

**AUTHORITIES FROM HTAi**

1. Rabia Sucu, HTAi President / TURKEY

2. Wija Oortwijn, HTAi Past President / THE NETHERLANDS

3. Ann Single, The Patient Voice Initiative / AUSTRALIA

**HTAi - SCIENTIFIC SECRETARIAT**

1. Manuel Espinoza, Chair, HTA Latin American HTA Policy Forum / CHILE

2. Andrés Pichón-Riviere / Director, Institute for Clinical Effectiveness and Health Policy (IECS) / ARGENTINA

3. Sebastián García Martí / HTA Department Coordinator, Institute for Clinical Effectiveness and Health Policy (IECS) / ARGENTINA

4. Federico Augustovski / Director, Institute for Clinical Effectiveness and Health Policy (IECS) / ARGENTINA

5. Valentina Stacco / Managing Coordinator, Projects, Institute for Clinical Effectiveness and Health Policy (IECS) / ARGENTINA

6. Alicia Powers / Events Coordinator, Health Technologies Assessment International (HTAI) / CANADA

7. Hana Price / Events Coordinator, Health Technologies Assessment International (HTAI) / CANADA
