# Supplementary material for: How can managed entry agreements contribute to coverage decisions in Latin America?
Source: Int J Technol Assess Health Care. 2024 Dec 2;40(1):e65. doi: 10.1017/S0266462324000527 (PMC11703624; doi:10.1017/S0266462324000527)
Supplement: García Martí et al. supplementary material 2 — García Martí et al. supplementary material [file S0266462324000527sup002.docx]

**How Can Managed Entry Agreements Contribute To Coverage Decisions In Latin America?**

**Full author names with affiliations**

Sebastián García Martí 1

Andrés Pichon-Riviere 1

Federico Augustovski 1

Manuel Espinoza 2

1. Institute for Clinical Effectiveness and Health Policy (IECS‐CONICET), Buenos Aires, Argentina
2. Department of Public Health, School of Medicine, Pontificia Universidad Católica de Chile, Santiago, Chile.

Running Title: Managed Entry Agreements in Latin America

**Corresponding author:** Sebastián García Martí (IECS) sgarciamarti@iecs.org.ar

Institute for Clinical Effectiveness and Health Policy, EMILIO RAVIGNANI 2024 (C1414CPV)

BUENOS AIRES - ARGENTINA

TEL/FAX: (+54-11)4777-8767

**ABSTRACT**

**Context:** Healthcare stakeholders in Latin America, including payers, manufacturers, and patients, seek to expedite access to technologies. However, uncertainty sometimes surrounds their true benefits and budgetary implications. Managed Entry Agreements (MEA) are proposed to address this uncertainty by redistributing risks among key actors.

**Objective:** The objective of Health Technology Assessment International's 2023 Latin American Policy Forum was to examine the potential utility of MEA in technology reimbursement and decision-making processes in the region.

**Methods:** This paper is based on a background document, survey, and the deliberative work of the country representatives and others who participated in the Policy Forum.

**Results:** Interest in MEA in Latin America is increasing, being financial agreements more prevalent than those based on clinical outcomes. During the Policy Forum potential barriers to MEA implementation were identified, such as the lack of legal frameworks, insufficient reliable data, and, in some cases, distrust among stakeholders. Some potential solutions were also identified, including early stakeholder involvement to enhance dialogue and understanding, and piloting shorter-duration MEA to facilitate the revision of agreement terms, especially in situations of epidemiological uncertainty.

**Conclusions:** The Policy Forum served as a valuable platform for discussing the importance of flexible MEA implementation that acknowledges data uncertainty, promotes transparent dialogue to incorporate opinions and values from all stakeholders, and develops legal frameworks to support effective technology access schemes in Latin America.

**Keywords**: Managed Entry Agreements, Risk Sharing, Financial, Technology Assessment, Latin America

**INTRODUCTION**

Healthcare stakeholders including payers, technology manufacturers, healthcare professionals, and patients share the goal of facilitating timely access to new technologies once they are market-approved. However, there can be uncertainty about the clinical effectiveness, safety, and/or cost-effectiveness of new treatments. The unpredictability of health system budget cycles can add financial risk and uncertainty to health systems^(^[[1]](#endnote-1)^)^, thereby limiting their ability to generate health and social value for the population. This situation merges important health system challenges, namely, financial risks that threaten system sustainability, and uncertainties in value creation that can lead to suboptimal efficiency.

Managed Entry Agreements (MEA), also named as Risk-Sharing Agreements (RSA), encompass different types of contractual arrangements that primarily redistribute financial risk between the contracted parties (payer and manufacturer). In these agreements, payers typically transfer some of the initial financial risk to the manufacturer, which can produce a more stable and sustainable pathway to provide coverage of new technologies. Other types of contracts (i.e., performance-based agreements) can be used to decrease risks in the production of health, helping to ensure the value for money spent on these new technologies.

MEA/RSA are increasingly used by health systems as tools to address challenges in access to innovative high-cost medicines. For example, in the United Kingdom, a number of approvals of new pharmaceuticals made by the National Institute of Health and Care Excellence (NICE) are conditional to some type of MEA/RSA^(^[[2]](#endnote-2)^)^ Other countries like France, Italy, and Sweden, have reported savings in their public health expenditures thanks to the implementation of MEA/RSA ^(^[[3]](#endnote-3)^)^. This global trend has drawn the attention of decision makers in many countries where they face balancing the demand for access to these new technologies challenges with the sustainability of the health system.

These agreements can be used to establish payment modalities and reimbursement conditions that are fair and equitable to both the health system payers as well as technology manufacturers, while ensuring access to quality care.

Two main MEA/RSA categories can be identified: financial-based agreements and those based on health outcomes^^[[4]](#endnote-4)^)^. Both aim to share risk between the payer and the manufacturer regarding technology reimbursement, but they address different areas of uncertainty. Financial agreements aim to reduce uncertainty about the budget impact of acquiring new health technologies, while performance-based agreements seek to reduce uncertainty regarding the effectiveness and cost-effectiveness (or performance) of innovations^(^[[5]](#endnote-5)^)^

Since 2016, Health Technology Assessment International (HTAi) has organized the Latin American Policy Forum (Policy Forum) to provide a neutral space for strategic discussions about the current state of health technology assessment (HTA), its development, and implications for the health system, industry, patients, and other stakeholders^(^[[6]](#endnote-6)^)^ ^(^[[7]](#endnote-7)^)^.

The 8th Latin American Policy Forum on Health Technology Assessment, held in Chile in 2023, addressed the topic, “How do new access schemes, including risk-sharing agreements, contribute to coverage decisions?”. The objectives were to: understand the current state of MEA/RSA in Latin America; to examine the potential utility of these agreements in the technology reimbursement process to facilitate patient access to technologies; to identify potential barriers and solutions to their implementation; and, to define a series of key principles to guide MEA/RSA implementation and use in the region.

This article does not represent a formal consensus nor necessarily a complete representation of participant perspectives. Rather, the intent of this paper is to summarize the discussions at the Policy Forum to provide valuable insights regarding the challenges and opportunities associated with the incorporation of MEA/RSA in the region.

**METHODS**

The Policy Forum scientific secretariat located at the Institute for Clinical Effectiveness and Health Policy prepared a background document summarizing the definitions and fundamentals of MEA/RSA, the types of instruments and their characteristics, potential uses, and challenges to incorporating these agreements into different HTA processes across the globe^(^[[8]](#endnote-8)^)^. The background document was informed by published and grey literature collected through an unstructured literature search based on recent key publications and discussions held with members of the Policy Forum organizing committee. The document provided general information about MEA/RSA to Policy Forum participants and harmonized definitions of key terms to facilitate discussions during the face-to-face meeting. It also presented the results of a survey administered to representatives of the 11 participating countries at the Policy Forum, which asked about the status of MEA/RSA utilization in HTA processes across in their country. It also included case reports written by local experts from three countries (Argentina, Brazil, and Uruguay) about their experiences with MEA/RSA.

The 8th Latin American Policy Forum was held in-person on 14 and 15 August 2023 in Santiago, Chile, with 44 participants (13 from HTA agencies; 6 from public, social security and private payers; 20 representatives from manufacturers of drugs, medical equipment, and diagnostic tests; 1 representative of the Pan American Health Organization (PAHO); and, 4 patient association representatives. Eleven (11) countries in the region were represented: Argentina, Brazil, Chile, Colombia, Costa Rica, El Salvador, Mexico, Paraguay, Peru, Dominican Republic, and Uruguay. HTAi representatives, academics, organizers and members of the scientific secretariat of the event were also in attendance. Annex I contains the list of participants, their affiliations, and countries.

The Policy Forum format included keynote presentations, breakout group sessions, and plenary discussions.

The aim of the first day was to introduce MEA/RSA from international and regional perspectives, and to explore how these mechanisms interact with HTA processes. Governmental representatives of countries in the region presented their experiences implementing MEA/RSA and the perspectives of participants from the pharmaceutical and device companies, as well as those of patient representatives, were shared.

Breakout groups were conducted following a discussion and debate methodology with practical exercises to identify barriers, threats, and potential solutions^(^[[9]](#endnote-9)^)^. Participants were divided into groups, maintaining in each a balance of countries and stakeholder representation. The results of the breakout group activities were presented and discussed in plenary sessions.

Over the two days of the event, two breakout group discussions were held. The first was to identify the potential benefits of the implementation of MEA/RSA in the region and to identify related barriers and facilitators. The second breakout group activity used case study analysis to explore the different potential activities and phases of such agreements along with suggested actions and guidance for successful implementation in each phase. The key points from the breakout groups were discussed during plenary, with a computerized voting system used to enable the ranking and prioritization of themes.

The Policy Forum was conducted under Chatham House Rules^(^[[10]](#endnote-10)^) (^[[11]](#endnote-11)^)^ and all materials were provided in both Spanish and English language.

**RESULTS**

Regional Survey

The survey about the status MEA/RSA implementation in the region was administered prior to the Policy Forum and responses were received from 17 participants from 11 countries. Seventy-five (75) percent reported use of some form of MEA/RSA in their country, with financial-based agreements more frequently used than those based on clinical outcomes. More than two thirds of respondents expressed interest in greater use in the future of agreements based on outcomes or value.

Regarding MEA/RSA implementation barriers, the survey revealed challenges related to the absence of legal frameworks to facilitate the use of these types of agreements, the lack of reliable data, and the mistrust that can at times be present among stakeholders. Some potential benefits of MEA/RSA use were also identified such as the generation of evidence regarding the effects and outcomes achieved with a technology, and opportunities for health system improvement in access and equity.

Annex II contains further detail of the survey results.

**Keynote Lectures and Stakeholders Presentations**

The Policy Forum included keynote lectures and stakeholder presentations that were discussed. In countries around the globe, it is predominantly financial MEA/RSA that are used, accounting for over two-thirds of all agreements. MEA/RSA are instruments to manage financial risk by distributing it in different configurations between the contracting parties. MEA/RSA can also play a strategic role in price negotiations, especially where the terms of pricing and discount agreements remain confidential.

The issue of fragmented health systems was also addressed, and it was suggested that centralization – both within countries as well as among countries – could enhance purchasing power and facilitate these types of agreements. It was recognized, however, that such strategies might be more effectively implemented in the private sector where it is easier to maintain the confidentiality of unit prices, which is in contrast with the complexities of reference pricing used in the public sector.

Several recommendations were made to optimize the implementation of MEA/RSA. Emphasis was placed on the need for these agreements to be tailored to specific contexts, as there is broad diversity of products and health systems. It was also mentioned that before considering the use of a MEA/RSA, a robust HTA needs to be conducted that takes into account the areas of uncertainty, particularly those related to clinical outcomes.

Furthermore, expanded dialogue among stakeholders, and the development of supportive regulatory frameworks, were proposed to facilitate the execution of MEA/RSA. HTA agencies were pointed to as key agents to potentially coordinate the complex interaction between payers and providers to streamline the MEA/RSA process. The importance of simplicity in developing solutions was highlighted, advocating for a space where the shortcomings and complexity of existing models can be openly explored and improved upon, instead of searching for a “magic bullet” solution to the complex challenges posed by MEA/RSA. Alternatively, the use of pilot agreements was suggested that would allow for the reassessment of the data requirements for MEA/RSA. A pragmatic approach was recommended for outcomes-based agreements, with consideration to the resourcing requirements in terms of monitoring and follow-up capacities in the health system.

**Breakout Group Activities**

The first breakout group activity of the Policy Forum aimed to identify the potential benefits in advancing in the use of MEA/RSA in the region and to identify possible barriers (and solutions) to their implementation.

The potential benefits identified by the groups are listed in Box 1.

**Box 1. Potential benefits associated with MEA/RSA implementation in the Latin American region**

- Allow for enhanced budget control, alongside improved health system planning and predictability.
- Contribute to healthcare savings by promoting spending efficiency and health system sustainability.
- Broaden more timely access to new and innovative technologies across the entire target population.
- Strengthen stakeholder involvement and foster trust, for example, with patients, and between the private and public sectors.
- Support the generation of additional (local) evidence including long-term data through robust outcome assessments.
- Enable the identification of pertinent questions for the design of pragmatic studies.
- Encourage the proper use of technology by care providers, with manufacturers motivated to ensure correct usage of their product to demonstrate improvements to patient management and care processes.
- Reduce uncertainty in cost-effectiveness estimates by leveraging real-world data.
- Decrease the tendency toward judicialization in healthcare decisions.

The possible benefits of MEA/RSA identified in the breakout groups were compiled and a vote was held in plenary in order to rank them. Forty-eight (48) Policy Forum participants voted for their top three ranked items (see Figure 1).

The top three voted potential benefits to MEA/RSA implementation were: improving access to technologies (whether early access or expansion of coverage to a larger population) that received 30 votes; the possibility to produce real-world data and evidence that received 24 votes; and, supporting health system spending efficiency and sustainability that received 20 votes.

**Figure 1. Potential benefits of MEA/RSA implementation in the Latin American Region – Ranked results (n=48)**


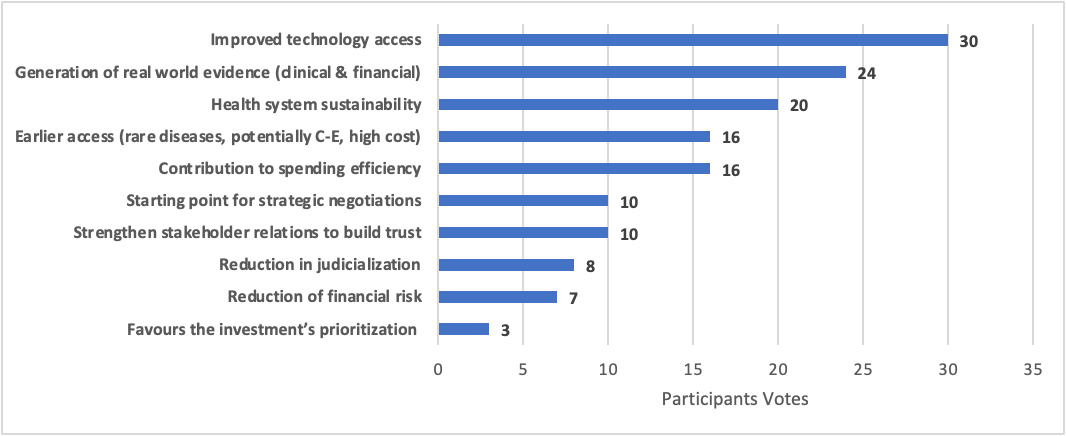


During the breakout group activity potential barriers to MEA/RSA implementation were also identified and these are presented in Box 2. The last three points listed describe barriers that are more relevant to outcomes-based agreements. The breakout groups also discussed potential solutions to overcome the identified barriers, and these are listed in Box 3.

**Box 2. Potential barriers related to MEA/RSA implementation in the Latin American region**

- The absence of a suitable legal framework, though is not a definitive barrier, can complicate the use of these agreements.
- Disagreements over price and the variation in purchase prices within fragmented health systems.
- Challenges in synchronizing health system budget cycles, which are often shorter than the agreement duration.
- Shortage of epidemiological data, such as precise information on incidence, prevalence, and rates of 'long responders', and the necessity to define target populations.
- Obstacles in engaging all relevant stakeholders (e.g., patients), and sometimes a lack of trust between public and private sectors.
- Poorly trained human resources (negotiation, technical skills, etc.).
- Vulnerability to economic or policy fluctuations that can impact agreement stability.
- The potential for agreement dilution if effective monitoring or predetermined exit strategies are not established.
- The need to clarify those who are responsible for agreement implementation, which may necessitate the involvement of third parties (such as scientific societies or members of assessment committees).
- Increased complexity in various areas, including the need for infrastructure, heightened administrative responsibilities, data collection and safeguarding, outcome monitoring, data management, ethical considerations, and training of personnel.

**Box 3. Potential solutions to overcome the identified barriers to implementing MEA/RSA in the Latin American region**

- Develop a legal framework or utilize existing frameworks, and advocate for regional guidelines that foster common agreements.
- Engage all relevant stakeholders and enhance communication, ensuring patients’ values are considered when selecting outcomes.
- Implement transparency standards to ensure diverse stakeholder participation throughout all phases of the process, openly presenting both benefits and drawbacks.
- Simplify agreements for ease of implementation, such as by limiting the number of treatment centers, targeting small populations, or selecting standardized outcomes.
- Forge agreements only when there is a practical likelihood of successful implementation.
- Conduct pilot programs with shorter durations to then reassess agreement terms, particularly under conditions of epidemiological uncertainty.
- Partner with academic institutions to achieve more accurate estimations at the initiation and conclusion of contracts.
- Form an audit team, comprising academic and third-party evaluators, to assess the process of indicator measurement.
- Establish comprehensive databases that include information from regulatory bodies.
- Enhance the management of patient data to ensure privacy and security.
- Increase awareness by showcasing good practices.

During plenary discussion of the benefits, barriers, and implementation strategies of MEA/RSA identified in the breakout groups, the importance of hearing diverse stakeholder perspectives was underscored as a crucial step towards putting these agreements in practice. It was also observed that while these agreements may not always reduce uncertainty, they can enable the redistribution of associated risks across the contracting parties.

Moreover, it was acknowledged that stakeholder engagement and dialogue is essential to gauge the varying perceptions of uncertainty and to ascertain at what level the degree of uncertainty could complicate decision-making processes. The experiences of international HTA agencies implementing early dialogue mechanisms may be worth examining in the near future, as they may provide useful insights about how to improve interactions between contracting parties^^[[12]](#endnote-12)^^.

During the Policy Forum’s second breakout group activity, case studies of MEA/RSA use were presented. Participants were asked to prioritize the different phases of MEA/RSA implementation in terms of their importance and complexity of implementation. The participants also identified actions and other suggestions to support an appropriate implementation of each of the phases.

The MEA/RSA phases presented to the breakout group participants were as follows:

1. Assessment of the health problem and disease burden
2. Assessment of the relevance and opportunity to use a MEA/RSA
3. Involvement of the different stakeholders
4. Negotiation and contractual framework between the parties
5. Implementation
6. Evaluation

Figure 2 presents the results of the classification of the phases according to importance and complexity for each of the four breakout group discussions. The Y-axis is the reported level of *importance* and the X-axis that of *complexity*.

The breakout groups converged in their classification of the ‘Negotiation and contractual framework’ phase (number 4 in the figure) as one of the most important yet most complex to implement. The phases of ‘Assessment of the health condition and disease burden’ and ‘Evaluation’ were next in relation to importance and complexity of implementation.

**Figure 2. Classification of different phases of MEA/RSA implementation regarding their *importance* and *complexity* by each of the four breakout groups**


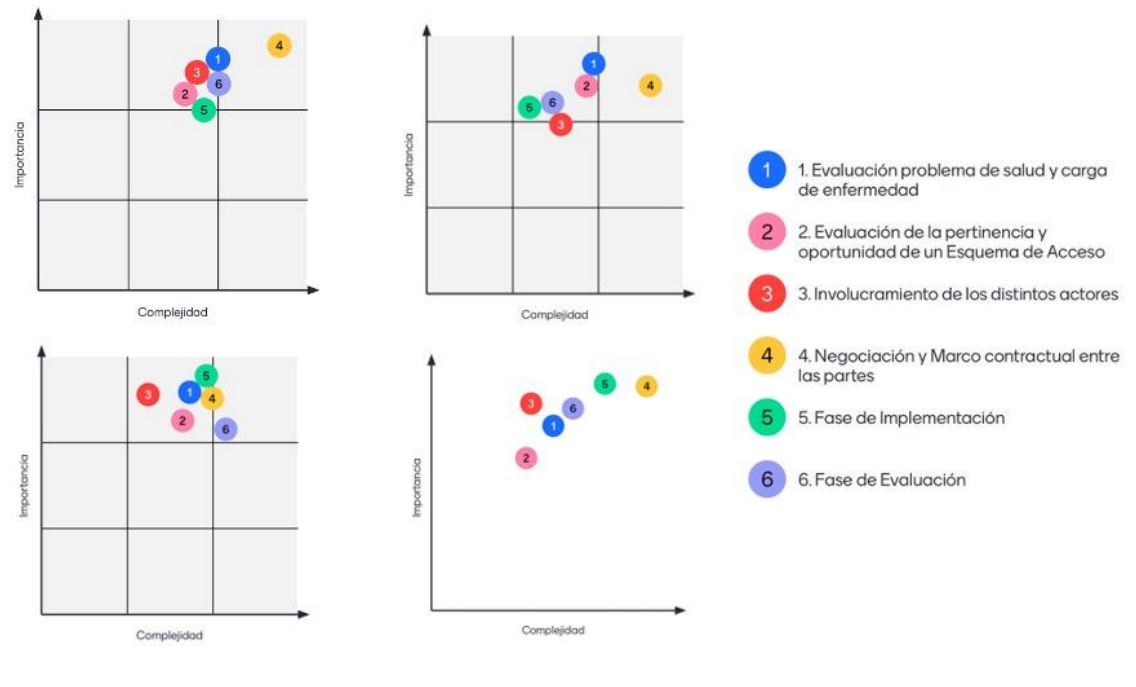


1. **Assessment of the health problem and disease burden**
2. **Assessment of the pertinence and opportunity for an access scheme**
3. **Stakeholder involvement**
4. **Negotiation and contractual framework between parties**
5. **Implementation**
6. **Evaluation**

During the breakout group discussions, several points were made regarding the significance and characteristics of different phases, as summarized below:

- Characterizing the health condition may be sometimes difficult because of scarce availability of information within the country. There are uncertainties faced in countries across the region concerning both health system costs and disease burden or outcomes.
- The success of negotiations relies on the representatives of the contracting parties. There is a need for specialized training in negotiation techniques to ensure successful MEA/RSA implementation, but access to such training is often lacking.
- A robust initial phase, coupled with a clear and all-encompassing agreement, can simplify the implementation process.
- It was suggested that countries consider building partnerships to facilitate collective purchasing.

Breakout group participants also identified several activities and other suggestions to consider in each MEA/RSA phase to support proper implementation, as follows:

**Phase 1. Assessment of Health Condition and Disease Burden**

- Recognizing the importance of the health condition and disease burden assessment phase is crucial; stakeholders must prioritize obtaining relevant local data.
- The lack of information should not be a barrier to move forward (explore the creation and use of registries).
- Engage a diverse group of stakeholders to ensure comprehensive problem characterization and to address demand.
- Determining the need for a MEA/RSA should be done during the HTA to streamline subsequent processes.

**Phase 2. Relevance of a MEA/RSA**

- Consensus and willingness to engage in MEA/RSA by all stakeholders are foundational to initiating the process.
- Financial departments in the health system should be involved early to align objectives.
- Established rules and frameworks can provide guidance on when to proceed with a MEA/RSA (e.g., budget thresholds for schemes based on clinical outcomes).
- Explore international collaborations for joint procurement.
- If high clinical uncertainty, such as is often the case for rare diseases, there is a need to monitor the market pipeline and conduct horizon scanning to enable advance planning for MEA/RSA.

**Phase 3. Stakeholder Involvement**

- Broad stakeholder mapping is essential, which should encompass payers, manufacturers, clinicians, patients, and legal experts.
- Clearly define a core set of relevant stakeholders and understand their needs and positions, with consideration to manage conflict of interest.
- Foster ongoing stakeholder dialogue and transparently manage expectations and information flows over the duration of the agreement.
- Consider involving a neutral third party to impartially assess MEA/RSA implementation.

**Phase 4. Negotiation and Contractual Framework**

- The negotiation phase is pivotal for successful implementation and evaluation of these agreements.
- Utilize contract frameworks or templates to streamline negotiations.
- Emphasize negotiation training, particularly regarding legal nuances, to build trust and foster mutual learning.
- Prepare for variable scenarios and maintain contract flexibility to adapt to emerging evidence or scenario changes (e.g. inflation or increased utilization).
- Ensure governance and conflict of interest policies are clear.
- Design agreements that integrate seamlessly into the clinical pathway.

**Phase 5. Implementation**

- Ensure robust information systems are in place with a realistic activity schedule for effective evidence management.
- Maintain a focused approach with a dedicated team and clearly defined outcomes.
- Proper planning in earlier stages leads to a smoother implementation phase, and this phase must be extremely rigid and controlled (for example in one or only two centers).

**Phase 6. Evaluation**

- Establish clear evaluation timelines, standards, and indicators.
- Encourage independent evaluations and stakeholder involvement.
- Consider the feasibility of renegotiation from the outset, particularly for complex agreements.
- Implement ongoing evaluation practices throughout the agreement's term.
- Decide on the public availability of data, including the potential use of real-world evidence.

During the plenary discussion held after the breakout group activities, the heterogeneity in nomenclature was observed with general agreement that it would be beneficial and important to standardize terminology and advance towards consensus on key concepts.

At the end of the Policy Forum, participants identified some lessons learned and future guidance for the appropriate implementation of MEA/RSA in the Latin American region. A preliminary list was produced in plenary, which was then read aloud and modified to include participants feedback and inputs to reach consensus.

**Lessons Learned from Policy Forum 2023 – Managed Entry Agreements/Risk Sharing Agreements**

- The creation of innovative access mechanisms should be linked with HTA processes. To achieve this, health systems must promote actions to strengthen HTA in the countries of the region.
- Based on international and regional experiences, it is recognized that there is value in implementing innovative access mechanisms in the region's health systems since they have the potential to optimize treatment access and contribute to health system sustainability.
- Every country encounters distinct technical, legal, and political hurdles when seeking to implement these agreements, which can complicate their execution. Nonetheless, regional experiences indicate that with sufficient will, and in the absence of explicitly prohibitive legislation, pathways to implement such agreements can indeed be established.
- It is important to promote collaboration both among countries in the region and with multilateral organizations, for example, the Pan American Health Organization, to share successful experiences.
- The preparation of this type of agreement should be accompanied by input collected from several stakeholders to put in place a structure that minimizes the risk of failure.
- Several challenges in the development of these agreements were recognized, including the need for greater harmonization of terminology and concepts, as well as the particular phases of a MEA/RSA. Countries should advance in defining reference frameworks for the preparation and implementation of these agreements. Regional collaboration is valued and promoted as a means for greater harmonization.
- A key facilitator in the implementation of these agreements is the transparency of the agreement processes.
- The publication of experiences using MEA/RSA in various countries should be encouraged.

**Conclusions – Key Messages**

The 2023 Policy Forum highlighted a growing interest in applying managed entry agreements (MEA) or risk sharing agreements (RSA) within the health technology assessment (HTA) processes and coverage decision making across Latin America.

The presentations made at the Policy Forum showed that there are experiences with such agreements in the region, noting a higher prevalence of financial-based over clinical outcomes-based MEA/RSA, which is similar to other regions^^[[13]](#endnote-13)^^ ^^[[14]](#endnote-14)^^.

Opportunities were recognized to broaden the implementation of MEA/RSA within the countries represented at the Policy Forum, with potential advantages to this including enhanced access to technologies (earlier and for a broader population), generation of real-world data to fill evidence gaps, improved budgetary efficiency, and promoting sustainability and equity within health systems.

Some MEA/RSA implementation barriers in the region were identified, including the absence of supportive legal frameworks or structures, inadequate availability of epidemiological and resource use data, distrust among stakeholders, and insufficient training to engage in such agreements. Additionally, the misalignment of MEA/RSA implementation with health system budgetary cycles poses a further challenge to their effective adoption and execution.

Potential solutions to address these barriers were also identified. These include the development of regulatory frameworks and regional guidelines to establish common agreements, the involvement of all relevant stakeholders throughout the process to enhance dialogue and transparency, and the formulation of agreements that prioritize implementation feasibility and enforceability. Additionally, it was suggested to undertake pilot agreements with short time horizons to allow for periodic review, particularly in contexts of epidemiological uncertainty. Initiating engagement earlier in the technology lifecycle, for instance, to establish patient registries and conduct early dialogue with regulatory bodies, was also noted as a potential solution to the challenges of MEA/RSA implementation.

Fragmentation and segmentation of health systems in the region was identified as a key issue that may affect the implementation of MEA/RSA. In countries or health systems with centralized purchasing processes, the implementation of MEA/RSA could be more easily utilized.

The importance of strengthening national and local HTA bodies in Latin America was highlighted, as MEA/RSA rely largely on the outputs of the HTA processes. HTA bodies could also implement horizon scanning systems to remain informed of emerging technologies and to undertake early dialogues with manufacturers about new access opportunities.

Maintaining the simplicity of MEA/RSA solutions was advised, acknowledging the inherent challenges and complexities in executing outcomes-based agreements. It was suggested that HTA bodies could be instrumental in the coordination of manufacturers and payers, and the inclusion of various stakeholders, including patients, was underscored as essential.

The development of legal structures was seen as crucial for advancing effective access to technology through MEA/RSA. A final remark for successful implementation of these agreements was the critical need for transparent dialogue, with emphasis on the necessity for clear and honest communication among stakeholders with distinct goals.

In conclusion, the Policy Forum served as a vital conduit for the exchange of insights and experiences about the use of MEA/RSA. It revealed that there are successful experiences using these types of agreements in Latin America, which may encourage other neighbouring countries to make progress to implement them in the short term. Policy Forum participants also emphasized the need for adaptable execution strategies for MEA/RSA to account for data uncertainties and they advocated for open discussions to understand diverse stakeholder perspectives.

**Acknowledgements**

Thank you to the event participants for their valuable contributions (List of participants in Annex I) and a very special thanks to Valentina Stacco, Policy Forum Secretariat, and the members of the Policy Forum Organizing Committee: Adriana María Robayo de IETS-Institute of Health Technology Assessment, Colombia; Luciene Bonan de CONITEC, Ministry of Health, Brazil; Fernanda Laranjeira of Medtronic, Brazil; Diego Guarín de Merck & Co., United States; William Dorling of PFIZER LIMITED, United States; Felipe Vera, Ministry of Health, Chile; Graciela Fernandez of the Fondo Nacional de Recursos, Uruguay; and Alicia Granados Sanofi, Spain.

**Author contributions**

SGM led the writing of this article. SGM, FA, APR and ME were involved in the activities’ design, implementation, and analysis. SGM, FA, APR and ME reviewed, improved, and approved this manuscript.

**Sources of Funding**

The organization of, and participation at, the Forum was funded by Health Technology Assessment International (HTAi). The preparation of this manuscript was completed by the Institute for Clinical Effectiveness and Health Policy (IECS) with funding from HTAi.

**Conflict of interests**

ME received personal funding and support for attending meetings as Chair of the Latin-American Policy Forum at HTAi. The other authors declared no conflicts of interest.

**Annex I**

**Participant List**

We thank all Policy Forum participants for their interest, involvement, and contributions.

**NON-PROFIT ORGANIZATIONS**

1. Silvina Beatriz Benchetrit / Ministry of Health GCABA / ARGENTINA

2. Manuel Donato / Ministry of Health Argentina / ARGENTINA

3. Luciene Bonan / National Committee for Technology Incorporation (CONITEC) / BRAZIL

4. Silvana Kelles / Unimed / BRAZIL

5. Felipe Vera Chandia / Ministry of Health Chile / CHILE

6. Adriana María Robayo García / Institute for Health Technology Assessment (IETS) /

COLOMBIA

7. Lizbeth Alexandra Acuña Merchan / Cuenta de Alto Costo / COLOMBIA

8. Hugo Marín Piva / Caja Costarricense de Seguro Social (CCSSS) / COSTA RICA

9. Carlos Diaz Huerta / Instituto Mexicano Seguro Social (IMSS) / MEXICO

10. Verónica Gallegos / Centro Nacional de Excelencia Tecnológica en Salud (CENETEC) /

MEXICO

11. Pedro Galvan / Instituto de Investigaciones en Ciencias de la Salud (IICS) / PARAGUAY

12. Lely Solari / Instituto Nacional de Salud (INS) / PERU

13. Andrea Gimenez / Ministry of Health Uruguay / URUGUAY

14. Graciela Fernandez / Fondo Nacional de Recursos (FNR) / URUGUAY

15. Yesenia Diaz / Superintendencia de Salud y Riesgos Laborales (SISALRIL) / REPÚBLICA

DOMINICANA

16. Ricardo Ruano / Ministry of Health El Salvador / EL SALVADOR

17. Natalia Messina / Ministry of Health de Argentina / ARGENTINA

18. Héctor Castro / Pan American Health Organization, World Health Organization /

COLOMBIA

19. Pilar Nehuelhal / Ministry of Health Chile / CHILE

**PARTICIPANTES FROM FOR-PROFIT ORGANIZATIONS**

1. Daniel Campos, ABBVIE

2. Rogerio Afif, ABBVIE

3. Mohit Jain, BioMarin

4. José Thomas, BioMarin

5. Javier Garcia, Bristol Myers Squibb

6. Arely Lemus, Bristol Myers Squibb

7. Fabian Ochoa, Edwards Lifesciences

8. Alma Delia Carbajal, Eli Lilly

9. Joice Valentim, F. HOFFMANN-LA ROCHE AG

10. Virginia Becerra, Roche International Ltd.

11. Natalia Tassara, Johnson and Jonhson

12. María Camila Bustos, Johnson and Jonhson

13. Daniela Paredes, Medtronic

14. Juan Valencia, Medtronic

15. Cintia Parellada, MERCK & CO

16. Diego Guarin, MERCK & CO

17. Nicola Romanello, PFIZER LIMITED

18. Mac Mullen, Mercedes, PFIZER LIMITED

19. Rafael do Prado Souza, Sanofi

20. Antonio Dos Santos, Sanofi

**PATIENT REPRESENTATIVES**

1. Cecilia Rodriguez / Ministry of Health, Chile – Area of Citizen Participation / CHILE

2. Alejandro Andrade / Chilean Federation of Rare Diseases - FECHER / CHILE

3. Virginia Llera / Geiser Foundation / ARGENTINA

4. Eva María Ruiz de Castilla / Latin America Patient Academy LAPA

**INVITED SPEAKER**

1. Pilar Pinilla-Dominguez / NICE / UNITED KINGDOM

**AUTHORITIES FROM HTAi**

1. Rabia Sucu, HTAi President / TURKEY

2. Wija Oortwijn, HTAi Past President / THE NETHERLANDS

3. Ann Single, The Patient Voice Initiative / AUSTRALIA

**HTAi - SCIENTIFIC SECRETARIAT**

1. Manuel Espinoza, Chair, HTA Latin American HTA Policy Forum / CHILE

2. Andrés Pichón-Riviere / Director, Institute for Clinical Effectiveness and Health Policy (IECS) / ARGENTINA

3. Sebastián García Martí / HTA Department Coordinator, Institute for Clinical Effectiveness and Health Policy (IECS) / ARGENTINA

4. Federico Augustovski / Director, Institute for Clinical Effectiveness and Health Policy (IECS) / ARGENTINA

5. Valentina Stacco / Managing Coordinator, Projects, Institute for Clinical Effectiveness and Health Policy (IECS) / ARGENTINA

6. Alicia Powers / Events Coordinator, Health Technologies Assessment International (HTAI) / CANADA

7. Hana Price / Events Coordinator, Health Technologies Assessment International (HTAI) / CANADA

**Annex II**

**Pre-Forum survey of current situation in the region -- Results**

(17 responses from 11 countries)


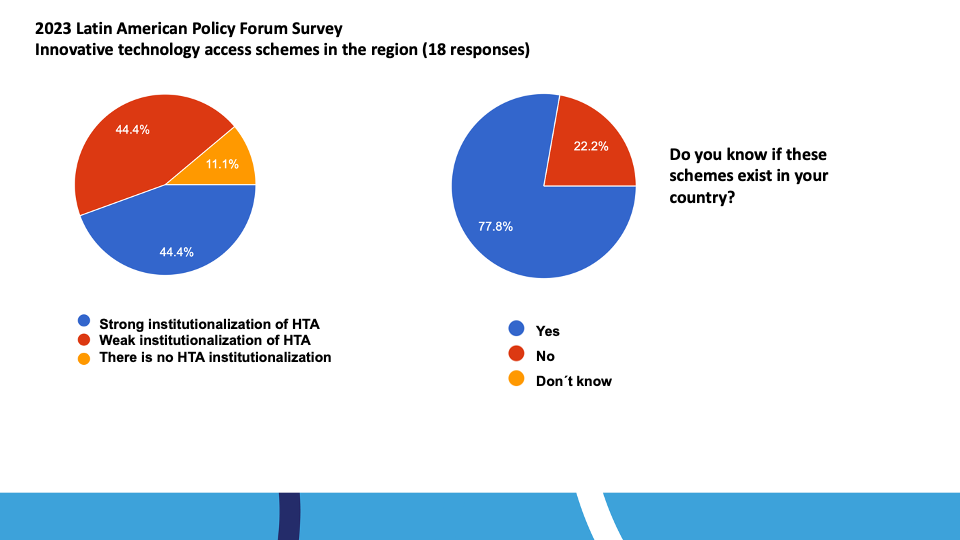


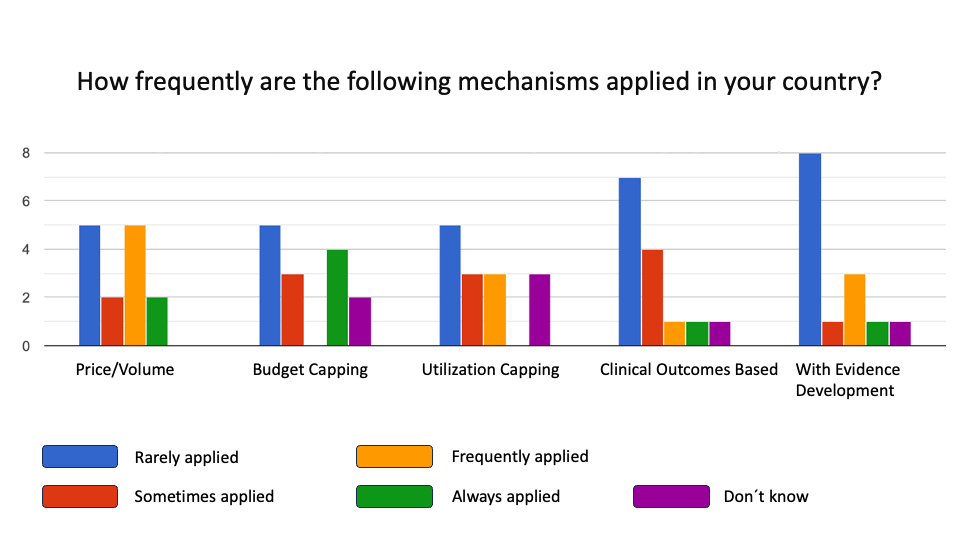


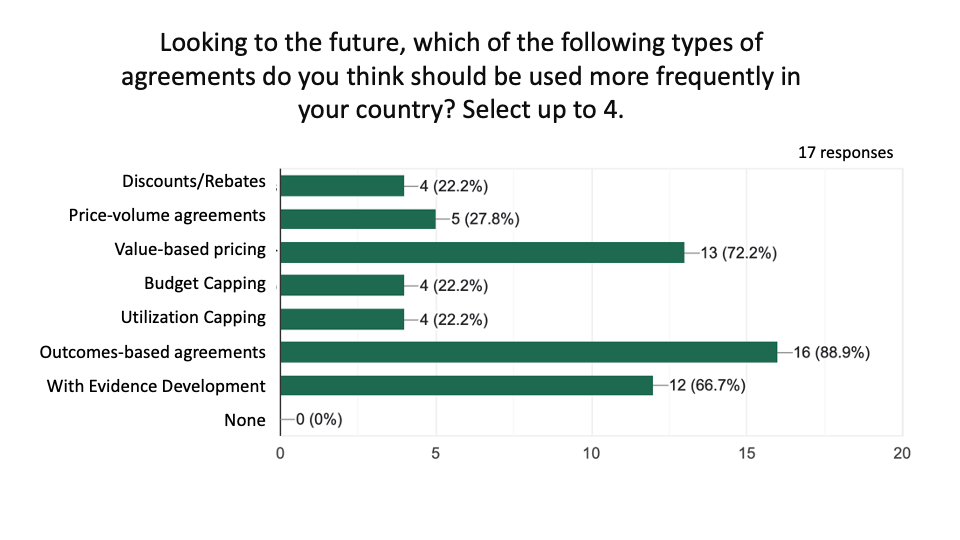


**References**

1. Hogervorst MA, Vreman R, Heikkinen I, et al. Uncertainty management in regulatory and health technology assessment decision-making on drugs: guidance of the HTAi-DIA Working Group. International Journal of Technology Assessment in Health Care. 2023;39(1):e40. doi:10.1017/S0266462323000375 [↑](#endnote-ref-1)
2. Managed Access. NICE. UK. Accessed at: <https://www.nice.org.uk/about/what-we-do/our-programmes/managed-access> [↑](#endnote-ref-2)
3. Wenzl, M. and S. Chapman (2019), "Performance-based managed entry agreements for new medicines in OECD countries and EU member states: How they work and possible improvements going forward", OECD Health Working Papers, No. 115, OECD Publishing, Paris. <https://doi.org/10.1787/6e5e4c0f-en> [↑](#endnote-ref-3)
4. Carlson, J. et al. (2010), “Linking payment to health outcomes: A taxonomy and examination of Health Policy, Vol. 96/3, pp. 179-190, <http://dx.doi.org/10.1016/j.healthpol.2010.02.005>. [↑](#endnote-ref-4)
5. Wenzl, M. and S. Chapman (2019), "Performance-based managed entry agreements for new medicines in OECD countries and EU member states: How they work and possible improvements going forward", OECD Health Working Papers, No. 115, OECD Publishing, Paris, <https://doi.org/10.1787/6e5e4c0f-en>. [↑](#endnote-ref-5)
6. Health Technology Assessment International (HTAi) [Internet] Latin America Policy Forum 2020. [cited 2021 Mar 17]. Available from: <https://htai.org/policy-forum/latin-america-policy-forum/> [↑](#endnote-ref-6)
7. Pichon-Riviere A, Soto N, Augustovski FA, Garcia-Marti S. Health Technology Assessment for Decision Making In Latin America: Good Practice Principles. Int J Technol Assess Health Care. 2018;34(03):241–7. [↑](#endnote-ref-7)
8. How can managed entry agreements can contribute to coverage decisions? VIII Latin America Health Technology Assessment Policy Forum (Latam Policy Forum). Chile, August 2023.

   Accessed at: https://past.htai.org/policy-forum/latin-america-policy-forum/background-papers/ [↑](#endnote-ref-8)
9. Johansson-Sköldberg U, Woodilla J, Çetinkaya M. Design Thinking: Past, Present and Possible Futures. Creat Innov Manag 2013;22(2):121–46. [↑](#endnote-ref-9)
10. Chatham House Rule | Chatham House [Internet]. [cited 2019 Jun 15]. Available from: <https://www.chathamhouse.org/chatham-house-rule> [↑](#endnote-ref-10)
11. 10 Brown T, Funk C. [Internet]. Design Thinking 2012 [cited 2019 Aug 23]. Available from: [www.hbr.org](http://www.hbr.org) [↑](#endnote-ref-11)
12. bargoyen-Roteta N, Galnares-Cordero L, Benguria-Arrate G, Chacón-Acevedo KR, Gutiérrez-Sepulveda MP, Low-Padilla E, De La Hoz-Siegler IH, Guevara-Pérez CI, Del Pozo-Pérez Á, Suárez M, Dauben HP, Otte M, Gutiérrez-Ibarluzea I. A systematic review of the early dialogue frameworks used within health technology assessment and their actual adoption from HTA agencies. Front Public Health. 2022 Oct 6;10:942230. doi: 10.3389/fpubh.2022.942230. PMID: 36276363; PMCID: PMC9583828. [↑](#endnote-ref-12)
13. Wenzl, M. and S. Chapman (2019), "Performance-based managed entry agreements for new medicines in OECD countries and EU member states: How they work and possible improvements going forward", OECD Health Working Papers, No. 115, OECD Publishing, Paris. [↑](#endnote-ref-13)
14. Ferrario, A. and P. Kanavos (2013), Managed entry agreements for pharmaceuticals: The European experience, EMiNet, Brussels. [↑](#endnote-ref-14)
